# In Vitro Evaluation of Increasing Avibactam Concentrations on Ceftazidime Activity against Ceftazidime/Avibactam-Susceptible and Resistant KPC-Producing *Klebsiella pneumoniae* Clinical Isolates

**DOI:** 10.3390/antibiotics12121707

**Published:** 2023-12-07

**Authors:** Marta Palombo, Benedetta Secci, Federica Bovo, Milo Gatti, Simone Ambretti, Paolo Gaibani

**Affiliations:** 1 Division of Microbiology, IRCCS Azienda Ospedaliero-Universitaria di Bologna, 40126 Bologna, Italyfederica.bovo@aosp.bo.it (F.B.); simone.ambretti@aosp.bo.it (S.A.); 2 SSD Clinical Pharmacology-Department, Integrated Management of Infectious Risk, IRCCS Azienda Ospedaliero-Universitaria di Bologna, 40138 Bologna, Italy; milo.gatti2@unibo.it; 3 Department of Medical and Surgical Sciences, Alma Mater Studiorum, University of Bologna, 40138 Bologna, Italy; 4 Department of Diagnostic and Public Health, Microbiology Section, Verona University, 37134 Verona, Italy

**Keywords:** βL-βLICs, KPC-producers, whole-genome sequencing, multidrug resistant

## Abstract

The novel β-lactam/β-lactamase inhibitor combinations (βL-βLICs) are one of the last-line resources available against multidrug-resistant (MDR) Gram-negative bacteria. Among βL-βLICs, ceftazidime/avibactam (CAZ-AVI) demonstrated strong activity against carbapenem-resistant *Enterobacterales* (CRE). Avibactam was proven to restore bactericidal activity of ceftazidime, inhibiting both KPC and OXA-48-like β-lactamases. Despite this, emergence of CAZ-AVI-resistant strains in *Enterobacterales* has been reported. Herein, we evaluated the in vitro ceftazidime activity in the presence of increasing concentrations of avibactam by the broth microdilution method against CAZ-AVI-susceptible and resistant genome-characterized KPC-producing *K. pneumoniae* (KPC-Kp) clinical isolates. Strains expressing KPC and co-expressing KPC/OXA-181 carbapenemase were selected on the basis of the different phenotypic traits for novel βL-βLICs and cefiderocol. Notably, avibactam at 8 mg/L maintained the MIC of ceftazidime above the clinical breakpoint in 14 out of 15 (93%) KPC-Kp resistant to CAZ-AVI. A high concentration of avibactam (i.e., 64 mg/L) is required to observe a bactericidal activity of ceftazidime against 9 out of 15 (60%) CAZ-AVI-resistant isolates. In vitro evaluation showed that with the increase in the concentration of avibactam, ceftazidime showed high activity against CAZ-AVI-susceptible strains. High concentrations of avibactam in vivo are required for ceftazidime to be active against CAZ-AVI-resistant KPC-Kp.

## 1. Introduction

During the last decades, the increasing rate of infections due to multidrug-resistant microorganisms (MDRs) has become a global public health problem [1,2,3] due to inadequate therapeutic options, resulting in the increase in mortality, morbidity, and higher healthcare-associated costs [4].

Carbapenems, and largely β-lactams, act as inhibitors of cell wall biosynthesis [3]. Carbapenems were generally used to treat infections sustained by *Enterobacteriales* species (such as *Escherichia coli* and *Klebsiella* spp.), producers of extended-spectrum β-lactamase (ESBL) and AmpC, which are usually associated with both community and hospital-acquired infections [5]. In this context, the emergence of carbapenem-resistant *Enterobacterales* (CRE) represents a relevant limitation in the therapeutic armamentarium available to treat severe infections in critically ill patients [6]. The World Health Organization (WHO) included CRE [7] among the most difficult to treat MDR organisms, for which the development of new antibiotics is needed [8].

Resistance to carbapenems is due to the production of carbapenemase enzymes [2]. Two distinct groups of carbapenemases exist, divided according to the catalytic mechanism that these enzymes use. Indeed, the active site can include a serine residue, a serine carbapenemase, [9] or metallic ions in the Metallo-β-Lactamases (MBL) [10]. Among the β-lactamases, the enzymes able to confer resistance to carbapenems belong to the Ambler Class A, B, and D. Among the Class A carbapenemases, the most worrisome enzyme is the *Klebsiella pneumoniae* carbapenemase (KPC); because of its association with *K. pneumoniae*, a microorganism correlates with high accumulation and transfer of resistance elements, and its usual location on self-conjugate plasmid [11]. Currently, KPC enzymes represent the most common resistance mechanism in different countries (United States, China, Israel, Greece, and Italy), where they are considered endemic [12]. The Class B carbapenemases are MBL proteins (mostly VIM, NDM and IMP), whereas Class D are OXA enzymes. Moreover, other additional resistance mechanisms may contribute to carbapenems resistance, e.g., lack of porin functionality and upregulation of the efflux system [6].

Due to the limited therapeutic options available to treat infections caused by MDR bacteria, old therapy options, such as polymyxins and fosfomycin, were re-evaluated [13]. At the same time, the development and valuation of novel therapeutic alternatives (e.g., tigecycline and carbapenems) have also been proposed [14,15]. In recent years, several novel β-lactam/β-lactamase inhibitor combinations (βL-βLICs) have been approved for treatment of infections sustained by Gram-negative bacteria, such as ceftazidime/avibactam (CAZ-AVI), meropenem/vaborbactam (MER-VAB), and imipenem/cilastatin/relebactam (IMI-REL) [7]. All of these new βLIs are effective against carbapenemase enzymes, thus restoring the bactericidal activity of the combined βL [8].

CAZ-AVI is the first component out of this novel generation of βL-βLICs. It was approved to treat complicated urinary tract infections (cUTIs) and complicated intra-abdominal infections (cIAIs) in 2015 and hospital-acquired and ventilator-associated bacterial pneumonia (HABP/VABP) in 2018 [16]. This drug combination consists of the antimicrobial agent ceftazidime, a third-generation cephalosporin associated with avibactam. Avibactam is a non-suicidal β-lactamase inhibitor, member of the diazabicyclo octane class (DBO). It is able to restore the in vitro bactericidal activity of third-generation cephalosporin compromised by the Class A (ESBL and KPC), Class C (AmpC), and Class D oxacillinase-48-like (OXA-48-like) β-lactamases [17]. Avibactam, at a concentration equal to 4 mg/L, has the ability to restore ceftazidime antimicrobial activity against 99% of *Enterobacteriales* strains according to the global surveillance study INFORM (International Network for Optimal Resistance Monitoring) [18]. Out of the novel βL-βLICs, the new meropenem–vaborbactam (MER-VAB) and imipenem–relebactam (IMI-REL) combinations were also approved to be used against infections caused by Gram-negative MDR pathogens for which there are limited therapeutic options [19]. Vaborbactam and relebactam are effective against Class A (ESBLs and KPCs) and Class C (AmpC) enzymes. None of the new βL-βLICs are effective against Class B (MBLs) enzymes [2].

Unfortunately, the emergence of strains producing Class A or D carbapenemase and resistant to the last generation of βL-βLICs has been recently described in [3,20,21,22]. It was reported that there is a rapid emergence of CAZ-AVI-resistant isolates in the United States and Europe [23,24]. Resistance to CAZ-AVI in *Enterobacteriales* was commonly related to different resistance mechanisms. The most common strategy observed involves modification on β-lactamase hydrolytic properties by specific mutations on Class A carbapenemase, specifically on KPC and CMY enzymes [25]. The most widespread mutations are amino acid substitutions located among the 164 and 179 positions of the aminoacidic sequence, the Ω loop. This is a conserved element involved in the structural arrangement of the binding cavity. In addition, two amino acids (*Glu* 166 and *Ans* 170) of the KPC Ω loop are directly implicated in the acylation and diacylation of the substrate. It was reported that mutation in this region can enhance the binding affinity between KPC enzymes and ceftazidime, resulting in a more efficient hydrolysis of this compound [26]. Since the clinical approval of CAZ-AVI by the FDA in 2015, different variants of the KPC protein have been available. Some KPC variants are able to increase the minimum inhibitory concentration (MIC) of CAZ-AVI to 128–256 mg/L; these are extremely higher values compared with the wild-type KPCs [27,28,29,30]. In addition to mutation on the *bla*_KPC_ gene, other resistance mechanisms are usually reported in *Enterobacteriales*. Among these, modifications in cell permeability by truncation or downregulation of porins and/or overexpression of efflux pumps may significantly contribute to CAZ-AVI resistance. Outer membrane proteins (OMPs) are an abundant component of Gram-negative bacteria. Porins typically aggregate to form pores, enabling the passage across the outer membrane of small hydrophilic compounds, such as β-lactams antibiotics. It was reported that truncated variants of Ompk35 and insertions in the L3 loop of OmpK36 are associated with resistance to CAZ-AVI [3]. It was largely reported that alterations in efflux pumps are critical for antibiotic resistance in Gram-negative bacteria. Resistance to carbapenems often correlates with the upregulation of *arcAB* and *aqxAB* genes in *K. pneumoniae* [3]. If specific mutations, such as the previously mentioned KPC variants, substantially increase CAZ-AVI MIC, the CAZ-AVI-resistant phenotype is commonly the outcome of varied resistance mechanisms, such as modifications of the antibiotic target or expression of an alternative one, alteration in outer membrane permeability, and antibiotic enzymatic inactivation.

Interestingly, it was recently shown in a preclinical model that increasing avibactam concentrations might allow for the resensibilization of ceftazidime against KPC isolates [10,31].

The aim of this study was to evaluate the in vitro activity of ceftazidime in the presence of increasing concentrations of avibactam (1, 2, 4, 8, 16, 32, and 64 mg/L) against 24 KPC-producing *K. pneumoniae* (KPC-Kp) including strains susceptible and resistant to CAZ-AVI.

## 2. Results

### 2.1. Phenotypic Characterization of K. pneumoniae Clinical Isolates

The antimicrobial susceptibility profiles of the 24 KPC-Kp clinical isolates susceptible and resistant to CAZ-AVI are summarized in Table 1. At the fixed avibactam concentration of 4 mg/L used to test the EUCAST clinical breakpoints of CAZ-AVI, 15 out of 24 (63%) KPC-Kp strains were found to be resistant and the other 9 (37%) isolates were found to be susceptible to CAZ-AVI. Moreover, 12 out of 24 (48%) KPC-Kp strains proved to be resistant to MER-VAB, 13 out of 24 (48%) were resistant to IMI-REL, and 16 out of 24 (64%) were resistant to CFD.

Notably, cross-resistance between CAZ-AVI and CFD was detected in 12 out of 15 CAZ-AVI-resistant (80%) KPC-Kp clinical isolates. Combined resistance to CZA-AVI and MER-VAB was identified in 9 out of 15 (60%) CAZ-AVI-resistant strains. Cross-resistance between CAZ-AVI and IMI-REL was observed in 10 out of 15 (67%) clinical KPC-Kp strains. In addition, 8 out of 15 (53%) CAZ-AVI-resistant clinical KPC-Kp strains proved to be resistant to CFD and IMI-REL, 9 out of 15 (60%) CAZ-AVI-resistant isolates showed resistance to MER-VAB and IMI-REL, and 7 out of 15 (47%) CAZ-AVI-resistant strains showed resistance to CFD, MER-VAB and IMI-REL. Notably, all CAZ-AVI-resistant *K. pneumoniae* co-producing KPC and OXA-181 showed resistance to all novel βL-βLICs.

### 2.2. Genomic Analysis of the Resistance Traits on K. pneumoniae Clinical Isolates

The genotypic resistance traits of KPC-Kp clinical strains included in the present study are summarized in Table 2. Multi-locus Sequence Typing (MLST) analysis showed that 12 out of 24 (50%) KPC-Kp strains belonged to sequence type (ST) 512, 5 (2%) to ST307, 5 (2%) to ST1519, and 2 out of 24 (0.2%) belonged to ST101 and ST528.

Genetic analysis established that all *K. pneumoniae* harboured the *bla_KPC_* gene and 10 out of 24 (42%) clinical strains co-harboured the *bla_OXA-181_* carbapenemase gene. In detail, analysis of carbapenemase genes showed that 12 out of 24 (50%) *K. pneumoniae* harboured *bla_KPC-3_*, 3 out of 24 (13%) harboured *bla_KPC-31_*, and 2 out of 24 (8%) harboured *bla_KPC-53_*, while the remaining strains harboured the *bla_KPC-148_*, *bla_KPC-49_*, *bla_KPC-130_*, *bla_KPC-68_*, *bla_KPC-125_*, and *bla_KPC_*_-*121*_ variants. Correlation between *bla_KPC_* variants and resistance to CAZ-AVI demonstrated that all of the 9 (100%) KPC-Kp susceptible to CAZ-AVI harboured the *bla_KPC-3_* variant, whereas 12 out of the 15 (80%) KPC-Kp resistant to CAZ-AVI carried a mutated KPC-3.

Analysis of the porin genes showed that 23 out of 24 (96%) KPC-Kp clinical isolates presented a truncated OmpK35, and the remaining strain (4%) showed a glutamine substitution with arginine at position 72 (Q72R). In addition, 16 out of the 24 clinical isolates (67%) possessed glycine (G) and aspartic acid (D) insertions at positions 135–136 (INS135GD) and 8 out of 24 (33%) possessed a truncated isoform of OmpK36.

### 2.3. Effects of Increasing Avibactam Concentrations on Ceftazidime MIC Observed in KPC-Producing K. pneumonia Clinical Isolates

**Figure 1:**
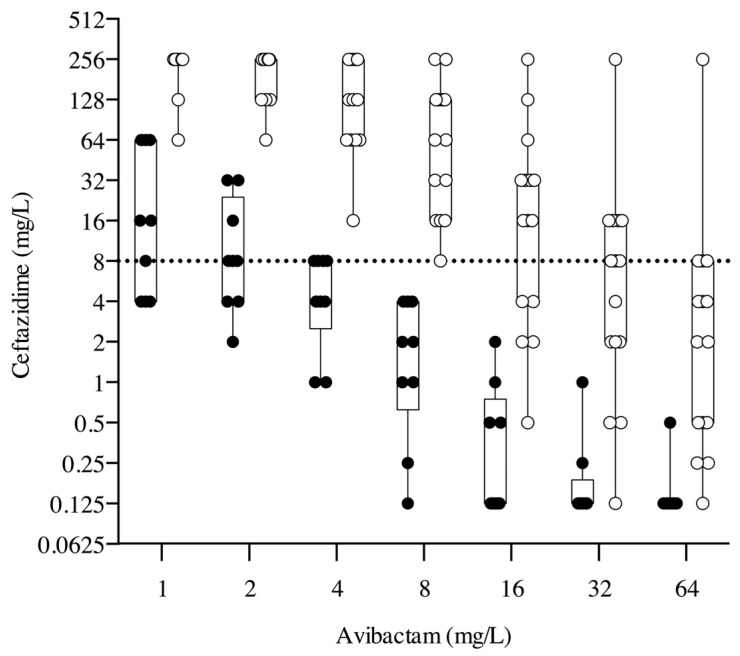
MICs of ceftazidime obtained with the broth microdilution method in presence of different concentrations of avibactam (1, 2, 4, 8, 16, 32, or 64 mg/L) against 24 KPC-producing *K. pneumoniae*. The values obtained for CAZ-AVI-sensitive and -resistant strains are reported in black and white, respectively. Dotted lines indicate CAZ-AVI breakpoint.

The values of ceftazidime MIC observed against the KPC-Kp isolates in the presence of increasing avibactam concentrations are depicted in Figure 1. Interestingly, both CAZ-AVI-susceptible and CAZ-AVI-resistant strains showed an almost log-linear decrease in ceftazidime MIC values in relation to the increase in avibactam concentrations. In particular, four and five out of the nine (44% and 67%, respectively) CAZ-AVI-susceptible KPC-Kp isolates exhibited an MIC of ceftazidime lower than the clinical breakpoint in the presence of avibactam concentrations equal to 1 and 2 mg/L, respectively. In addition, all CAZ-AVI-susceptible KPC-Kp isolates showed a 3.14, 16.36, 23.16, and 31.43-fold reduction in ceftazidime MIC when compared to an avibactam concentration of 4 mg/L and tested in the presence of avibactam at concentrations equal to 8, 16, 32, and 64 mg/L.

On the contrary, resensibilization to ceftazidime (MIC values equal to or below 8 mg/L) was observed in the CAZ-AVI-resistant strains in relation to the increase in avibactam concentrations above the fixed testing threshold of 4 mg/L. In detail, 14 out of 15 (93%) CAZ-AVI-resistant KPC-Kp isolates remained above the clinical breakpoint of ceftazidime in the presence of avibactam equal to 8 mg/L, 10 out of 15 (66%) isolates remained resistant to ceftazidime with avibactam equal to 16 mg/L, 6 out of 15 (40%) isolates remained resistant to ceftazidime with avibactam equal to 32 mg/L, and 1 out of 15 (7%) isolates remained resistant to ceftazidime with avibactam equal to 64 mg/L.

Notably, no differences were observed between *K. pneumoniae* clinical strains expressing KPC carbapenemase and co-expressing KPC/OXA-181 enzymes.

**Figure 2:**
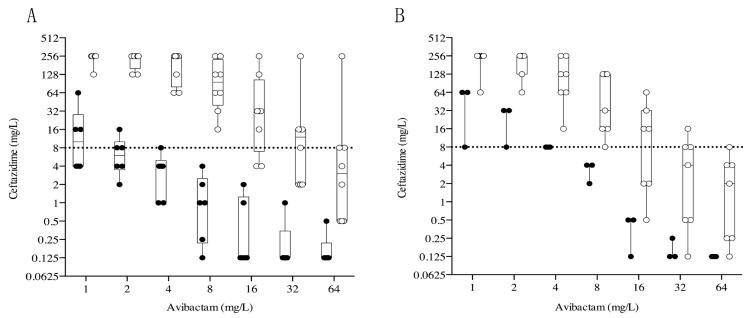
MICs of ceftazidime obtained with broth microdilution method in presence of different concentration of avibactam (1, 2, 4, 8, 16, 32, or 64 mg/L) against 14 KPC-producing (**A**) and 10 Kp-co-producing KPC/OXA-181 (**B**) *K. pneumoniae.* The values obtained for CAZ-AVI-sensitive and -resistant strains are reported in black and white rings, respectively. Dotted lines indicate CAZ-AVI breakpoint.

In detail, results of the ceftazidime/avibactam susceptibility test conducted on *K. pneumoniae* harboured only one carbapenemase gene (*bla*_KPC_). As we can see (Figure 2A) three out of six (50%) and five out of six (83%) CAZ-AVI-susceptible strains showed an MIC of ceftazidime lower than the breakpoint in the presence of avibactam concentrations of 1 and 2 mg/L, respectively. None of the eight CAZ-AVI-resistant isolates had an MIC of ceftazidime below than the clinical breakpoint (8 mg/L) when the avibactam concentration was equal to 8 mg/L. Avibactam at concentrations equal to 16, 32, and 64 mg/L reduced the MICs of ceftazidime below the clinical breakpoint in two out of eight (25%), four out of eight (50%), and seven out of eight (88%) of CAZ-AVI-resistant strains.

Analysis of *K. pneumoniae* co-producing the KPC and OXA-181 enzymes (Figure 2B) showed that one out of three (33%) CAZ-AVI-susceptible isolates had an MIC of ceftazidime lower than the clinical breakpoint in the presence of avibactam equal to 1 and 2 mg/L. When ceftazidime was tested with increasing the concentration of avibactam in this study, that is, more than the canonical 4 mg/L, one out of seven (14%) isolates reached the ceftazidime clinical breakpoint with avibactam equal to 8 mg/L, three out of seven (43%) with avibactam equal to 16 mg/L, six out of seven (86%) with avibactam equal to 32 mg/L, and seven out of seven isolates (100%) fell below the clinical breakpoint with avibactam 64 mg/L.

In summary, the ceftazidime MICs of CAZ-AVI-susceptible KPC-Kp strains were highly influenced by the avibactam concentration, independently of the co-expressing KPC and OXA-181 carbapenemase.

## 3. Discussion

Herein, we evaluated the in vitro activity of ceftazidime in the presence of different avibactam concentrations. Our findings showed that increasing the avibactam concentrations above the fixed threshold of 4 mg/L may increase ceftazidime activity against well-characterized KPC-Kp clinical isolates having different genotypic traits, both CAZ-AVI susceptibility and resistance.

Interestingly, CAZ-AVI-susceptible KPC-Kp showed consistent decrease in ceftazidime MIC at all tested avibactam concentrations. Conversely, most of the in vitro CAZ-AVI-resistant KPC-Kp isolates showed a progressive resensibilization to ceftazidime with avibactam ranging between 32 and 64 mg/L.

Based on these findings, it could be hypothesized that CAZ-AVI-susceptible and resistant strains could respond differentially to changing avibactam concentrations. In particular, it would be expected that different genotypic traits could influence the different responses to CAZ-AVI.

In this regard, administering ceftazidime–avibactam by continuous infusion (CI) may help to achieve higher and more stable avibactam concentrations in patients, potentially rendering it as a more effective treatment of infections sustained even against borderline susceptible KPC-Kp. It was showed that high-dose CI ceftazidime–avibactam was successful in microbiological eradication against a case series of infections caused by borderline CAZ-AVI-susceptible pathogens [13]. In a proof-of-concept study, it was shown that the microbiological outcome might have been influenced by the avibactam concentration levels in a case series of patients affected by CRE and treated with CI of ceftazidime–avibactam. Specifically, patients with lower avibactam steady-state concentration during treatment had a trend toward microbiological failure, likely due to less pronounced ceftazidime MIC reductions [14]. This may support the contention that achieving higher steady-state concentrations of avibactam would be helpful even for treating infections sustained by borderline CAZ-AVI-resistant KPC-Kp. Previous studies showed that administering full dose of CAZ-AVI by CI may grant average free avibactam concentrations up to 14.0 mg/L (7.5–17.0 mg/L) among the critically ill patients or even up to 24.8 mg/L (20.7–25.8 mg/L) among those undergoing continuous renal replacement [13].

Our study has some limitations. The number of clinical isolates analyzed was small and clinical data for featuring strains were unavailable. Conversely, the findings may indicate the importance that an in-depth knowledge of the PK/PD behaviour of CAZ-AVI in each single patient may have in maximizing treatment effectiveness of infections caused by KPC-Kp.

## 4. Materials and Methods

### 4.1. Bacteria Population and Phenotypic Characterization

In this study, we included 24 clinical strains of *K. pneumoniae* collected from patients recovered at the S. Orsola-Malpighi Hospital between March 2017 and September 2021. The isolates were selected based on antimicrobial susceptibility to novel βL-βLICs and CFD. Species identification was performed by means of matrix-assisted laser desorption/ionization time-of-flight (MALDI-TOF) mass spectrometry with MALDI Biotyper system (Bruker Daltonik, Bremen, Germany). The antimicrobial susceptibility profile was initially tested using the automated MicroScan Walkaway-96 system (Beckman Coulter, Brea, CA, USA). Subsequently, the values of MIC to CAZ-AVI, CFD, MER-VAB and IMI-REL were confirmed by means of gradient diffusion strip (GDS) (Liofilchem, Roseto degli Abruzzi, Italy). Briefly, each isolate was uniformly plated on Mueller–Hinton agar plate by a swab soaked in saline bacterial suspension arranged for each clinical isolate. The suspension was obtained by isolating colonies from 18 h agar plate suspended in saline solution (NaCl 0.9%) to obtain a turbidity equal to 0.5 McFarland (McF) standard. Above each plate, thus prepared, the strip of each antibiotic (CAZ-AVI, CFD, MER-VAB, and IMI-REL) was positioned. The plates are incubated for 16–20 h at 37 °C. The MIC values were interpreted according to the European Committee for Antimicrobial Susceptibility Testing (EUCAST) clinical break-points v12.0 (https://www.eucast.org/clinical_breakpoints/ (accessed on 1 January 2023). All clinical strains were screened for carbapenemase production during the routine workflow established at the Microbiology Unit of the S. Orsola-Malpighi Hospital. In detail, carbapenemase type was determined using MALDI-TOF for 11.109 *m*/*z* specific peak detection for KPC [11] and multiplex immunochromatographic (IC) assay NG Test CARBA 5 (NG Biotech, Guipry-Messac, France). The discordant results between MALDI-TOF specific peak and IC assays, and for CAZ-AVI phenotypes are analysed with molecular assay (Xpert Carba-R, Cepheid, Sunnyvale, CA, USA) to identify carbapenemase gene and to exclude MBLs [12].

### 4.2. Genomic Characterisation

Genomic characterisation of the 24 clinical strains included in the present study was performed as formerly described [20]. Briefly, genomic DNA was extracted by DNeasy Blood&Tissue Kit (Qiagen, Hombrechtikon, Switzerland), according to manufacturer’s instructions. Libraries were generated using DNA Prep Library Preparation Kit (Illumina, San Diego, CA, USA), according to manufacturer’s instructions. Sequencing was performed with Illumina iSeq100 platform (Illumina, USA), iSeq Reagent Kit v.2 with 2 × 150 paired-end reads was used. Paired-end reads quality was evaluated using FastQC software v0.12.01 (https://www.bioinformatics.babraham.ac.uk/projects/fastqc/, accessed on 9 October 2022). Assembly was performed by SPAdes v.3.15.4. MLST evaluation was executed by comparing each genome against typing schemes deposited in the PubMLST database (https://pubmlst.org/ (accessed on 9 October 2022)) using MLST v2.11 (https://github.com/tseemann/mlst (accessed on 9 October 2022)). Genomes annotation was implemented using RAST server (https://rast.nmpdr.org, accessed on 9 October 2022) [32,33,34,35,36]. The presence of β-lactamase determinants was evaluated against the Comprehensive Antibiotic Resistance Database (CARD) (https://card.mcmaster.ca, accessed on 8 October 2022) and the Beta-Lactamase-DataBase (BLDB) (http://bldb.eu, accessed on 9 October 2022), whereas porin genes were manually investigated using BLAST analysis against reference proteins (OmpK35 [O87753] and OmpK36 [D6QLX8]).

### 4.3. Antimicrobial Susceptibility Testing of Ceftazidime/Avibactam with Broth Microdilution Method

The in vitro ceftazidime susceptibility with the increase in concentrations of avibactam was assessed by broth microdilution method as previously described [20]. Briefly, each plate was arranged with a serial dilution of ceftazidime (1, 2, 4, 8, 16, 32, 64, 128, and 256 mg/L) and added with the increase in avibactam concentrations (1, 2, 4, 8, 16, 32, 64 mg/L). Bacteria inocula were prepared by isolating colonies from agar plate incubated for 24 h at 37 °C. Bacterial cells were suspended in saline solution (NaCl 0.9%) to obtain turbidity to 0.5 McF standard. The suspension was diluted in Mueller–Hinton broth so that it has a bacterial load of 10^5^ CFU/mL. Each sample was tested in triplicate. The MIC of ceftazidime was determined as the lowest ceftazidime concentration that can inhibit 50% of bacterial growth at each avibactam concentration. Each experiment was conducted in triplicate.

## 5. Conclusions

Our findings demonstrated that the increase in avibactam concentrations may increase the antibacterial activity of ceftazidime against KPC-Kp clinical isolates and that concentrations higher that 16 mg/L might even allow to overcome in vitro CAZ-AVI resistance. Based on these findings, we hypothesize that in vitro antimicrobial resistance to CAZ-AVI could not be the only determinant conditioning microbiological failure in patients under CAZ-AVI-based treatment. Administering CAZ-AVI by CI and measuring avibactam concentrations in real-life may be helpful tools for optimizing treatment with CAZ-AVI among the critically ill patients. Prospective confirmatory studies are warranted for assessing the role that a therapeutic drug monitoring guided therapy of CAZ-AVI administered by CI may have in improving the outcome of infections caused by KPC-Kp with borderline susceptibility or even with theoretical CAZ-AVI in vitro resistance.

## Figures and Tables

**Table 1:** Phenotypic characteristics of KPC-Kp included in this study.

Isolates	^a^ MIC (mg/L)			
	^b^ CAZ-AVI	^c^ MER-VAB	^d^ IMI-REL	^e^ CFD
Kp1	2	0.125	0.5	0.19
Kp2	4	0.25	0.5	**12**
Kp3	2	0.032	0.125	0.023
Kp4	4	0.50	1	0.25
Kp5	2	1	0.5	0.047
Kp6	0.5	0.032	0.25	0.064
Kp7	4	**32**	**4**	**6**
Kp8	6	**32**	**4**	**16**
Kp9	3	**32**	**4**	**8**
Kp10	**>256**	8	**4**	**24**
Kp11	**>256**	2	0.25	**4**
Kp12	**32**	**256**	**4**	**14**
Kp13	**>256**	**32**	**32**	**32**
Kp14	**64**	0.25	0.5	0.5
Kp15	**32**	1.5	0.5	**16**
Kp16	**>256**	3	0.25	**4**
Kp17	**>256**	0.047	0.19	**24**
Kp18	**64**	**48**	**4**	**24**
Kp19	**12**	**32**	**4**	2
Kp20	**>256**	**16**	**4**	**24**
Kp21	**>256**	**24**	**6**	**16**
Kp22	**>256**	**32**	**4**	**32**
Kp23	**12**	**12**	**6**	0.75
Kp24	**48**	**16**	**4**	**16**

The values of higher breakpoint are reported in bold. ^a^ Applying EUCAST breakpoint; ^b^ CAZ-AVI, ceftazidime–avibactam; ^c^ MER-VAB, meropenem–vaborbactam; ^d^ IMI-REL, imipenem–relebactam; ^e^ CFD, cefiderocol.

**Table 2:** Genotypic characteristics of KPC-Kp included in this study.

Isolates	MLST ^a^	*β*-Lactamase Enzyme	Multidrug Efflux Pumps Genes	Major Porins Mutations	
				OmpK35	OmpK36
Kp1	ST307	KPC-3	*emrD*, *oqxA*, *oqxB19*	truncated at aa 229	truncated at aa 134
Kp2	ST307	KPC-3	*emrD*, *oqxA*, *oqxB19*	truncated at aa 229	truncated at aa 182
Kp3	ST307	KPC-3	*oqxA*, *oqxB*	*Gln*72*Arg*	truncated at aa 182
Kp4	ST1519	KPC-3	*emrD*, *oqxA*, *oqxB*	truncated at aa 41	Ins135*GlyAsp*
Kp5	ST101	KPC-3	*emrD*, *oqxA*, *oqxB20*	truncated at aa 61	truncated at aa 134
Kp6	ST528	KPC-3	*emrD*, *oqxA*, *oqxB19*	truncated at aa 132	truncated at aa 182
Kp7	ST512	KPC-3	*emrD*, *oqxA*, *oqxB*	truncated at aa 41	Ins135*GlyAsp*
Kp8	ST512	KPC-3	*emrD*, *oqxA*, *oqxB*	truncated at aa 41	Ins135*GlyAsp*
Kp9	ST512	KPC-3	*emrD*, *oqxA*, *oqxB*	truncated at aa 41	Ins135*GlyAsp*
Kp10	ST1519	KPC-3	*emrD*, *oqxA*	truncated at aa 229	truncated at aa 134
Kp11	ST1519	**KPC-31**	*emrD*, *oqxA*, *oqxB*	truncated at aa 41	Ins135*GlyAsp*
Kp12	ST307	KPC-3	*emrD*, *oqxA*	truncated at aa 229	truncated at aa 134
Kp13	ST512	**KPC-53**	*emrD*, *oqxA*, *oqxB*	truncated at aa 41	Ins135*GlyAsp*
Kp14	ST1519	KPC-148	*emrD*, *oqxA*, *oqxB*	truncated at aa 41	Ins135*GlyAsp*
Kp15	ST512	**KPC-49**	*emrD*, *oqxA*, *oqxB*	truncated at aa 41	Ins135*GlyAsp*
Kp16	ST1519	**KPC-130**	*emrD*, *oqxA*	truncated at aa 41	Ins135*GlyAsp*
Kp17	ST307	**KPC-31**	*emrD*, *oqxA*, *oqxB19*	truncated at aa 229	truncated at aa 182
Kp18	ST512	**KPC-68**	*emrD*, *oqxA*, *oqxB*	truncated at aa 41	Ins135*GlyAsp*
Kp19	ST512	**KPC-66**	*emrD*, *oqxA*, *oqxB*	truncated at aa 41	Ins135*GlyAsp*
Kp20	ST512	**KPC-125**	*emrD*, *oqxA*, *oqxB*	truncated at aa 41	Ins135*GlyAsp*
Kp21	ST512	**KPC-121**	*emrD*, *oqxA*, *oqxB*	truncated at aa 41	Ins135*GlyAsp*
Kp22	ST512	**KPC-31**	*emrD*, *oqxA*, *oqxB*	truncated at aa 41	Ins135*GlyAsp*
Kp23	ST512	KPC-3	*emrD*, *oqxA*, *oqxB*	truncated at aa 41	Ins135*GlyAsp*
Kp24	ST512	**KPC-66**	*emrD*, *oqxA*, *oqxB*	truncated at aa 41	Ins135*GlyAsp*

The KPC variants associated with CAZ-AVI resistance are reported in bold. ^a^ MLST, Multi-locus Sequence Typing.

## Data Availability

Data are contained within the article.
